# Reduced age-wise disparity in estimated cervical cancer screening participation rates after applying hysterectomy correction: a population-based cross-sectional study

**DOI:** 10.1186/s12905-026-04414-1

**Published:** 2026-03-26

**Authors:** Yuba Raj Paudel, Linan Xu, Se-Inyenede Onobrakpor, Hana Dampf, Huiming Yang, James A. Dickinson, Gary Teare, Kamala Adhikari

**Affiliations:** 1 Cancer Prevention and Screening Innovation, Primary Care Alberta, Calgary, Canada; 2 School of Public Health, University of Alberta, Edmonton, Canada; 3 Provincial Screening Programs, Primary Care Alberta, Calgary, Canada; 4 Public Health Surveillance and Informatics, Alberta Health Services, Edmonton, Canada; 5 Department of Family Medicine, Cumming School of Medicine, University of Calgary, Calgary, Canada; 6 Department of Community Health Sciences, Cumming School of Medicine, University of Calgary, Calgary, Canada

**Keywords:** Cervical cancer screening, Hysterectomy, Hysterectomy-correction, Pap tests, Disparities

## Abstract

**Introduction:**

Cervical cancer screening participation rates (CCSPR) are often underestimated when women who have undergone total hysterectomy for benign reasons remain in the screening-eligible population. This study aimed to improve the accuracy of Alberta’s CCSPR by excluding these individuals and to assess disparities in participation across sub-populations.

**Methods:**

We conducted a retrospective cross-sectional study using provincial administrative health data. Women aged 25–66 years were included, and those with a documented total hysterectomy for benign indications were excluded from the screening-eligible population. We stratified the population into two age strata (25–49 and 50–66) while estimating screening participation rate by zone of residence, migration status, and ethnicity. We conducted multivariable modified poisson regression to estimate hysterectomy corrected prevalence ratios for being uptodate with cervical cancer screening.

**Findings:**

A total of 1,324,927 women aged 25–66 year at index date were included in the analysis. Correction for hysterectomy increased CCSPR by 3% point among women aged 25–66 years (uncorrected: 57.3%, corrected 60.4%). Upon correction, age for peak participation shifted from 30–39 years to 40–59 years. After hysterectomy correction, those aged 25–29 years and 60–66 years showed lower screening participation compared to those aged 30–59 years. North Zone continued to show lower participation even after correction for hysterectomy (55.2%) compared to Edmonton (62.9%) and Calgary (61.8%) in the 25-49-year age group. Inter-provincial migrants (55.8%) and international migrants (58.8%) had lower screening participation than non-immigrants (67.0%). Similarly, the Asian population (57.6%) and the Black population (60.2%) showed a lower screening participation compared to the White (62.0%) and the Hispanic population (63.1%). Multivariable modified Poisson regression analysis confirmed significantly lower likelihood of up-to-date screening among the youngest (25–29) and the oldest (60–66) age groups, residents of the North zone, and immigrants particularly those identifying as Asian or Black.

**Conclusion:**

Applying hysterectomy correction increased Alberta’s estimates for screening participation. The uncorrected cervical cancer screening participation rate shows a declining trend by increased age (after 30–39), but this trend is no longer apparent after hysterectomy correction. Future interventions and patient education need to consider other key factors (zone of residence, migration status, ethnicity) including age to design targeted interventions.

**Supplementary Information:**

The online version contains supplementary material available at 10.1186/s12905-026-04414-1.

## Background

Cervical cancer incidence declined in Canada over the past decades due to the implementation of organized screening programs and other preventive strategies [1]. However, the incidence has increased in recent years in Canada since 2015 [1]. Despite being one of the most preventable and treatable forms of cancer, more than 1,500 Canadian women are projected to receive a diagnosis of cervical cancer in 2023 [1]. Regular cervical cancer screening, early diagnosis and management of abnormal cell changes and HPV vaccination help to prevent cervical cancer. However, non-participation in cervical cancer screening continues to drive much of the disease burden [2, 3].

The Alberta Cervical Cancer Screening Program (ACCSP) currently estimates cervical cancer screening participation rate (CCSPR) by dividing the number of women who received at least one cytology test in last 3 years (up to 42 months) in the province by total eligible population [4]. ACCSP has a provincial target of 80% participation [4, 5]. However, screening participation has declined in the past decade (68% in 2011 and 64% in 2017). Although CCSPR has returned to pre-pandemic level after COVID-19 disruptions (62% in 2021),substantial disparities persists largely in rural zones: North, Central, and South zones reporting lower CCSPR compared to urban Zones like Edmonton and Calgary [4]. This underscores the need for targeted, equity-focused interventions to improve participation rates.

Accurate estimation of CCSPR is essential for public health planning and equitable intervention design. A key methodological concern is the inclusion of women who have had a total hysterectomy for benign reasons, who are no longer eligible for cervical cancer screening. Failure to exclude this group from the denominator results in underestimation of true participation rates [6–9].Together, these programmatic and methodological improvements are critical for enhancing participation rates and identifying sub-populations for targeted interventions.

Hysterectomy is a common surgical procedure. According to Alberta Health Services records, approximately 4,000 hysterectomies were conducted in Alberta in 2022/23 [10].Although considered to be an overestimation, self-reported data suggest hysterectomy prevalence in 2008 was 15.3%, 24% and 39.4% among those aged 40–49 years, 50–59 years and 60–69 years, respectively [11]. The annual hysterectomy rate among the female population in Alberta was 317 per 100,000 in 2023 [12], with rates varying across AHS zones. Calgary (273/100,000) and Edmonton (294/1000,000) have the lowest hysterectomy rates whereas Central (470/100,000), North (403/100,000) and South zone (376/100,000) have a higher rate. Adjusting for this substantial hysterectomy prevalence and accounting for variation by age category, geography and population groups is essential to accurately estimating CCSPR.

The indication for hysterectomy and the type of procedure performed have important implications for continued cervical cancer screening. Women who have had a subtotal hysterectomy (uterus removed but cervix retained), as well as those who received a total hysterectomy (removal of both the uterus and cervix) due to cervical cancer or high-grade precancerous lesions, still require appropriate screening and follow-up [13, 14]. In contrast, women who underwent a total hysterectomy for benign conditions do not need further cervical cancer screening, as evidence suggests that potential harms outweigh any benefits [15, 16]. Despite this, many studies do not exclude hysterectomized women when estimating CCSPR [17, 18]. Adjusting these estimates by removing women with total hysterectomies—referred to as hysterectomy correction—can lead to an increase in CCSPR [19].

Only a few Canadian studies have reported hysterectomy-corrected cervical cancer screening participation rate [7, 9, 20]. Snider et al. conducted a Pan-Canadian study and reported CCSPR correcting for self-reported hysterectomy prevalence [7]. Their study used data from a survey conducted in early 90’s and self-reported hysterectomy prevalence may be overestimated. In a randomly selected cohort of approximately 12,500 people aged 20–69 years from British Columbia, Tseng et al. estimated a CCSPR in 2008–2010 correcting for hysterectomy prevalence based on administrative data [20]. However, hysterectomy prevalence was reported to be quite low (3.7%) in their sample population, possibly not reflecting a true population prevalence. Forte et al. reported corrected estimates for CCSPR in 2006–2008 for selected Canadian provinces but the source of hysterectomy data and the methods for hysterectomy correction were not clearly mentioned. Therefore, a study based on more recent Canadian data systematically investigating the hysterectomy corrected CCSPR in a province-wide population using a comprehensive and reliable hysterectomy prevalence data is lacking.

Accurate measurement of screening participation rates will help understand the current screening participation in general population and across sub-populations and inform knowledge users in furthering the efforts to improve cancer screening rates. ACCSP has been estimating hysterectomy corrected CCSPR using discharge abstract database (DAD) and National ambulatory care reporting system (NACRS) to identify women with hysterectomy from 2003 and onwards. Expanding these data sources to include all available administrative database (DAD, NACRS as well as Physician claims database) was essential to identify hysterectomies conducted in Alberta from earliest years of data availability. Additionally, procedure codes and billing codes have changed over time, making it crucial to use time-specific coding to accurately identify hysterectomies from the earliest data available date until index date. Furthermore, data on disparities in CCSPR among different sub-populations based on migration status, and ethnicity to inform prevention strategies was limited. Identifying such differences is critical for guiding equitable public health outreach and policy.

### Objectives

This study objectives were two-fold.

1. To estimate age-specific hysterectomy corrected cervical cancer screening participation rate in Alberta,
2. To assess variation in hysterectomy corrected cervical cancer screening participation by geographic regions, migration status and ethnic backgrounds in Alberta.

We conducted this study in partnership with ACCSP to refine the measurement of CCSPR. ACCSP is Alberta’s provincial program that works to increase cervical cancer screening participation with the aim of reducing cervical cancer incidence and mortality. We involved decision-makers and program managers from ACCSP throughout the study to understand measurement issues and collaboratively develop refinement to support sustainable implementation. The improved measurement of CCSPR will enable ACCSP to effectively identify screening gaps on an ongoing basis.

## Methods

### Study design

We conducted a population-based, retrospective cross-sectional study using a linked dataset obtained from the multiple administrative databases in Alberta. The study index date was July 1, 2019, and screening status was assessed over 42-months following index date (July 1, 2019-December 31, 2022).

### Setting

The Alberta Cervical Cancer Screening Program (ACCSP) is a provincial population-based screening program, which was initiated in 2000 [21]. In accordance with 2016 ACCSP clinical practice guideline(CPG), women and people with a cervix aged 25–69 are recommended to undergo free-of-charge cervical cancer screening with Pap tests every three years [15]. ACCSP sends screening invitation letters to age eligible women. Women book the appointments by telephone or email with their primary care providers to undergo screening. Primary care providers/physicians provide screening services, communicates results and next steps to patients. The ACCSP acts as a safety net to communicate results and sends reminder letters for women who are overdue for cervical cancer screening [22]. Additionally, Primary care providers initiate opportunistic discussions and offer screening services to eligible women when they present for other health concerns. The ACCSP also recommends continued annual vaginal vault smears for people who have had a total hysterectomy due to biopsy confirmed high grade lesions. ACCSP has been estimating CCSPR following Canadian Partnership Against Cancer’s recommended approach [4]. Aggregate estimates for women aged 25–69 years who had a hysterectomy (identified from DAD and NACRS using procedure codes) or have a prior history of cervical cancer were removed from the denominator and the numerator.

### Study population

**Fig. 1:**
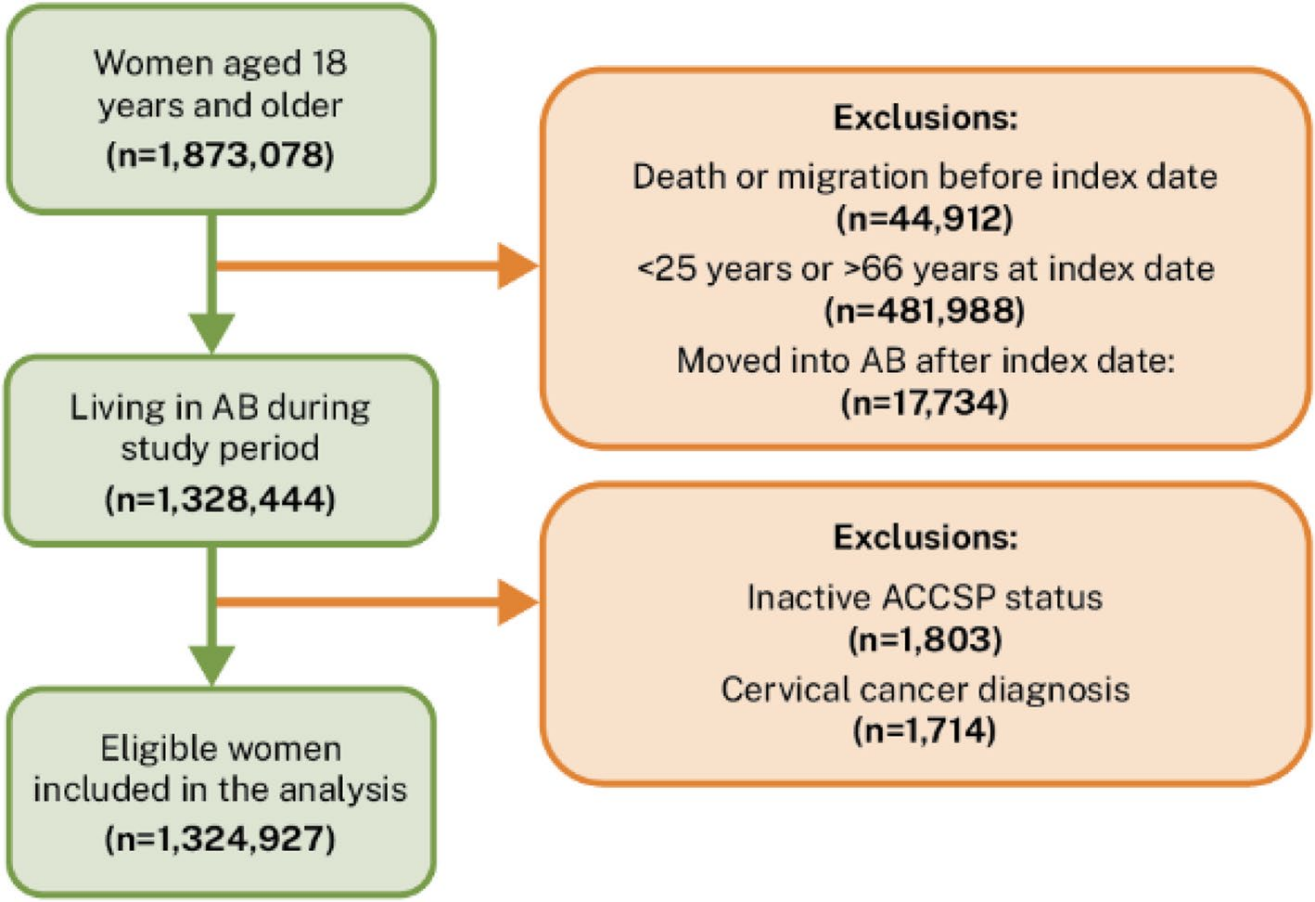
Study population selection flow chart

The cohort included women aged 25–66 years at the index date, ensuring all participants remained within the screening-eligible age range throughout follow-up. We excluded individuals with missing age, sex, or postal code; those with a history of cervical cancer; women who explicitly requested to be excluded from the ACCSP; in these cases, their data are not used in any analysis or reporting, demographic ineligibility, including women who have moved out of Alberta, are deceased or presumed deceased, or are no longer covered by Alberta Health insurance (e.g., military service) or; or who moved into Alberta after the index date (i.e., < 42 months of residency during follow-up). The study population selection process is shown in Fig. 1.

### Data sources and linkage

We used AHCIP registry to identify eligible population for this study. AHCIP has demographic information (i.e., age, sex) for all Albertans registered in the publicly funded healthcare system. The Alberta Cervical Cancer Screening Program (ACCSP) database was used to identify women who received Pap tests. The Alberta Cancer registry was used to identify women who had a cervical cancer diagnosis before the index date. The Alberta Cancer Registry (ACR) is a population-based registry that records topography, morphology, behaviour and cancer related information of Albertans diagnosed with cancer from early 1990 s and onwards. We used DAD, NACRS, and physician claim databases to identify hysterectomy procedures conducted in the province. DAD includes data on discharges from all hospitals in Alberta. NACRS has data on ambulatory care visits and procedures (e.g. day surgery, emergency room visits, community rehabilitation services. Physician claims database includes data on physician/practitioner submitted billing claims for services provided on a fee- for- service basis for reimbursement to the provincial government. Immigrant registry/database was used to identify women who immigrated to Alberta from 1994 until 2022. This dataset contains information on country/province of origin, date of migration, age at migration. We used postal code translation files for geocoding participants’ residential location. We deterministically link databases using scrambled PHNs (unique identifiers created using individual’s PHNs). The database linkage process is shown in Additional file 1.

### Variable definition

#### Outcome

Screening participation rate was calculated as a proportion of eligible women aged 25–66 years of age who received at least one pap test during the 42 months. Pap tests were identified through the cervical cancer screening program database and physician billing codes (13.99BA,13.99BC). Cervical cancer screening status was classified as:

- Currently Up-to-Date (CUTD): If women had a Pap test (or colposcopy exam) within the target 42 months. If women had multiple pap test (or colposcopy exam) within the target 42 months, the first exam was selected. Colposcopy exam was included in the CUTD estimation because women who had abnormal pap test findings in the previous 42 months and currently are under colposcopy follow-up are not expected to receive routine screening Pap tests during the follow-up period. The follow up period typically lasts approximately three years. Therefore, these women are still considered up to date with respect to cervical cancer screening. ACCSP uses this approach to measure CCSPR.
- No Record of Screening (NRS): If women had no record of a Pap test (or colposcopy exam) ever between January 1 st, 2012, and December 31, 2022.
- Overdue: By default, after inclusion in either CUTD or NRS categories, the remaining women were defined as overdue.

During analysis, CUTD were considered one category and NRS and overdue categories were combined to form Not Up-To-Date category.

#### Hysterectomy correction

We used ICD 10 procedure codes to identify hysterectomies from national ambulatory care reporting system (NACRS) and discharge abstract database (DAD) from 2003 until 2019; and used health services intervention codes in Physician claims database (Additional file 2) to identify hysterectomy procedures conducted in the province from 1994 until 2019. These procedure codes are widely used procedure codes and have been validated in Canadian setting [23]. Time-specific codes were applied to account for changes in classification systems over time (see Additional file 2). Women with prior hysterectomy due to high-grade cervical pathology were retained in the denominator. As direct clinical data were unavailable, a proxy definition was used: if a woman continued receiving screening after hysterectomy, she was presumed to have a history of high-grade lesion in line with ACCSP guidelines recommending annual vault smears.

### Main predictor variables

- Age group: 25–29, 30–39, 40–49, 50–59 and 60–66 years.
- Zone of residence: South, Calgary, Central, Edmonton, North.
- Migration status: international migrants, inter-provincial migrants, non-migrants.
  o Immigrants who migrated to Alberta between 1994 and 2019, lived in Alberta for at least 3 years, and whose place of origin was a country or jurisdiction from outside Canada were classified as international immigrants.
  o Immigrants who migrated to Alberta between 1994 and 2019, lived in Alberta for at least 3 years, and who migrated from other Canadian provinces and territories were classified as interprovincial immigrants.
  o Remaining populations, by default, were considered non-immigrants.
- Ethnicity: White, Black, Hispanic, and Asian. Ethnicity was predicted based on the last name using Rosenman et al.’s algorithm [24]. The surname list was validated by comparing the predicted ethnicity against race/ethnicity using census surname list in the US population which can be considered similar to the Canadian population in terms of ethnicity composition. The predicted race/ethnicity from the surname list used in their study showed more than > = 95% correlation for White, Black, Hispanic and Asian ethnicities against the prediction using census surname list [24]. We ensured data anonymity by limiting access to surname data to the analyst who prepared data for analysis so that researchers who analysed the data only had access to predicted ethnicity data.

Covariates included:

- Social and material deprivation index (in quintiles): 1 representing women from least deprived quintile and 5 representing most deprived quintile.
- Urban–rural residence status (7 category scale): representing metro areas to rural and remote areas.

### Analysis

We conducted this study to refine the measurement of CCSPR with a practical and reproducible method to exclude women who have had a hysterectomy due to benign diseases from the eligible population. ACCSP analytic team co-led this study to refine the methods of routine reporting. We tested various approaches/data sources including survey and administrative data, regression-based methods and lifetable-based method to estimate hysterectomy prevalence (results are available upon request). However, considering the complexity of some of these methods and practicality of implementing them in routine reporting of the performance by screening program, we focused our analysis and results based on administrative data to identify women who had a record of total hysterectomy in the past 25 years for this study. Consequently, the current analysis approach was anticipated to generate actionable findings to inform efforts aimed at reducing inequity in screening uptake across sub-populations.

We used screening data for 42 months following the index dates to analyze screening participation (July 2019-December 2022). To calculate hysterectomy corrected CCSPR, we first identified women who had a total hysterectomy before the index date. Using proxy definition for “hysterectomy due to benign reasons”, we ensured that women with total hysterectomy due to benign indications within the past 25-years were individually excluded from the eligible population. Age standardized screening participation rate was calculated using the 2016 Canadian female population as the standard. We estimated age-specific screening participation in 5 age categories. We calculated uncorrected CCSPR and corrected CCSPR by age categories and calculated the difference to compare relative impact of correction.

We stratified our population into two broad age categories, 25–49 and 50–66 years for investigating variation of corrected CCSPR based on zone, migration status and ethnicity. This stratification was important for potential under correction of hysterectomy data in the 50–66 years category. We conducted multivariable modified poisson regression to estimate screening participation prevalence ratios. Modified Poisson regression is an alternative to logistic regression, where the parameters are risk ratios or prevalence ratios (PR) rather than odds ratios. It is a generalized linear model with a linking function, the logarithm of the probability of a binary event instead of the log odds [25]. We ran two separate models for 25–49 years and 50–66 years. We included social and material deprivation index, urban/rural place of residence as well as four predictor variables (age category, zone of residence, migration status and predicted ethnicity) in both models. Robust error variance and associated 95% CI were estimated.

Additionally, to compare corrected CCSPR for women aged > 50 years using hysterectomy prevalence based on administrative data, we derived hysterectomy prevalence from Canadian Community Health Survey (CCHS) 2013–14 (Additional file 3) and estimated corrected CCSPR using the formula proposed by Beavis et al. [26]. This data source is used by other provincial health authorities for correction and provides a prevalence-based method to compare with the individual history method used in this analysis.

## Findings

A total of 1,324,927 women aged 25–66 years were included in this analysis. Of these, 6.7% had a total hysterectomy before the index date (July 1, 2019). Hysterectomy prevalence increased with age, from < 1% among those aged 25–29 years to 13% among those aged 50–60 years and 12.5% among those aged 60–66 years. After excluding women who had a hysterectomy for benign conditions (n = 70,174, 5.3%), 1,254,753 were considered eligible population for cervical cancer screening (corrected denominator). Therefore, 1,324,927 was considered the denominator for uncorrected estimates and 1,254,753 was considered the denominator for the corrected estimates.

### Population characteristics

Nearly three-quarter of our study population (74%) were aged between 30–59 years and a similar proportion (73.3%) lived in Edmonton and Calgary zones (Table 1). Compared to women aged > = 50 years, those aged < 50 years were more likely to be international and inter-provincial immigrants (57.7% vs 35.9%), while older women (> = 50 years) were more likely to live in rural and remote areas (20.2% vs17.1%). Higher proportion of women aged > = 50 years (84%) were White compared to 25–49 years (80%).

**Table 1:** Characteristics of study population

	25–49 years (*n* = 837,981)		50–66 years (*n* = 416,772)	
Variables	**Frequency**	**Percent**	**Frequency**	**Percent**
Age category				
25–29	169,481	20.22	N/A	N/A
30–39	376,777	44.96	N/A	N/A
40–49	291,723	34.81	N/A	N/A
50–59	N/A	N/A	258,246	61.96
60–66	N/A	N/A	158,526	38.04
Zone of residence				
South	50,138	5.98	27,686	6.64
Calgary	342,937	40.93	165,931	39.81
Central	78,622	9.38	46,747	11.22
Edmonton	276,615	33.01	134,024	32.16
North	89,649	10.7	42,377	10.17
Place of residence				
Metro	489,140	58.37	225,365	54.07
Moderate Metro Influence	105,051	12.54	60,563	14.53
Moderate Urban Influence	179,18	2.14	9,215	2.21
Urban	82,524	9.85	37,556	9.01
Rural	95,869	11.44	59,958	14.39
Rural Central Area	30,569	3.65	16,416	3.94
Rural remote	16,890	2.02	7,692	1.85
Pampalon social deprivation index				
Q1 (least deprived)	131,487	15.69	80,708	19.37
Q2	137,508	16.41	65,609	15.74
Q3	157,362	18.78	74,872	17.96
Q4	174,334	20.8	86,084	20.65
Q5 (Most deprived)	204,473	24.4	92,781	22.26
Pampalon material deprivation index				
Q1 (least deprived)	167,785	20.02	79,423	19.06
Q2	157,749	18.82	76,839	18.44
Q3	156,392	18.66	79,090	18.98
Q4	158,834	18.95	84,696	20.32
Q5 (Most deprived)	164,404	19.62	80,006	19.2
Immigration status				
International immigrants	203,709	24.31	54,662	13.12
Inter-provincial immigrants	279,378	33.34	94,981	22.79
Non-immigrants	354,894	42.35	267,129	64.09
Ethnicity				
White	667,139	79.65	350,088	84.08
Hispanic	38,740	4.63	15,976	3.84
Black	29,178	3.48	10,137	2.43
Asian	100,488	12.0	39,437	9.47
Others	2,003	0.24	731	0.18

*N/A* Not applicable

### Impact of hysterectomy correction on CSSPR

**Fig. 2:**
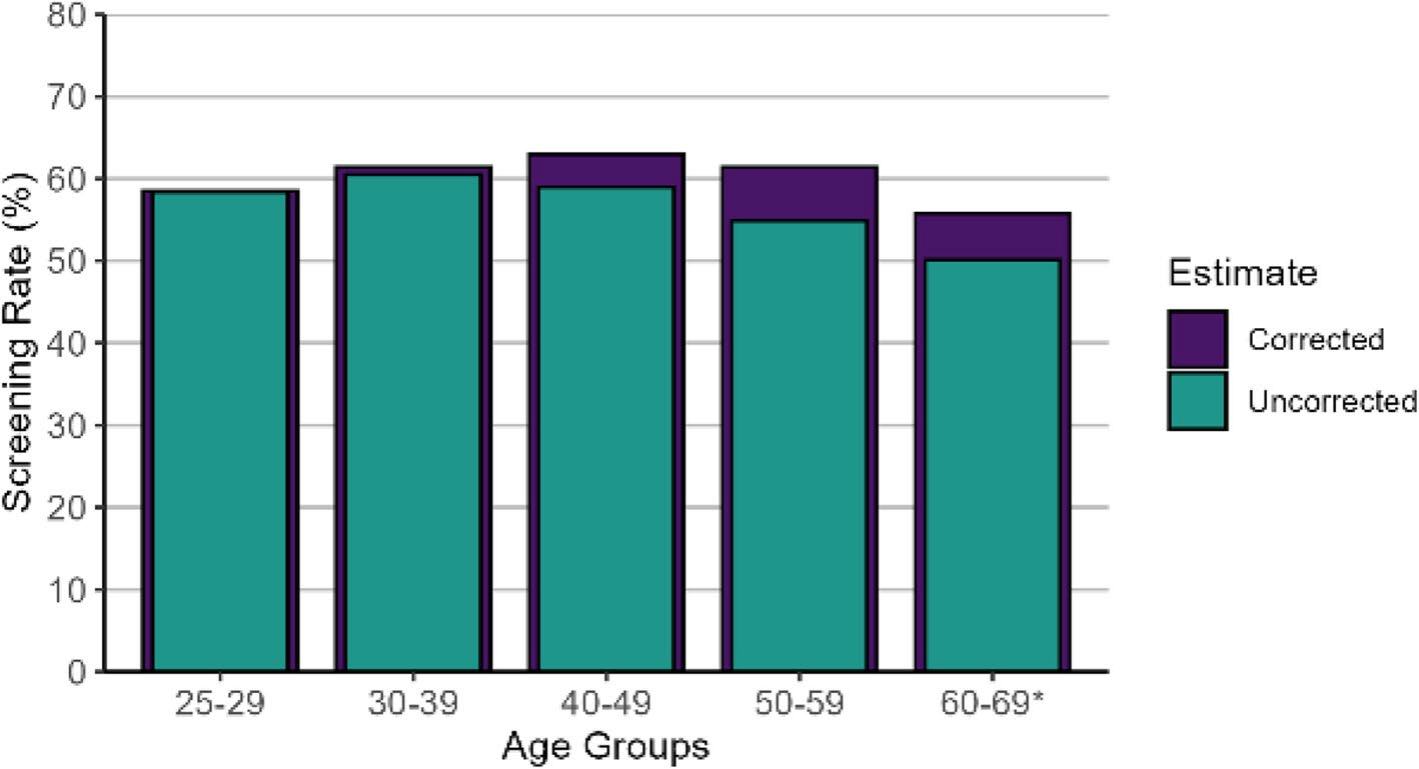
Screening participation rate (corrected and uncorrected for hysterectomy) by age groups *although maximum age at index date in the study population was 66 years, maximum age at screening was 69 years

Age standardized CCSPR increased by more than 3% point, from 57.3% (uncorrected) to 60.4% (corrected) in our study population. The difference between uncorrected and corrected CCSPR was < 1% point in 25–39 years, 4% point in 40–49 years and approximately 6% point in 50–66 years (Fig. 2). As expected, the impact was higher among age groups with higher prevalence of hysterectomy. Before hysterectomy correction, screening participation was the highest in the 30–39 age group (60.5%), with lower rates observed in the 25–29 age group (58.4%) and those aged 40 and above (< 60%) (Fig. 2). After hysterectomy correction, screening participation increased in the 40–49 (63%) and 50–59 (61.4%) age groups, eliminating the decline seen after age 40. Corrected CCSPR remained similar between 30–60 years of age but was markedly lower among women aged 60–66 (55.8%).

### Geographic disparities

Our results show that, prior to hysterectomy correction, there was a 3–8% point difference in screening participation between Central, North and South zones compared to Edmonton and Calgary zones among those aged 25–49 years (Table 2). After hysterectomy correction, the disparity across zones was reduced to < 2 points in South (61.4%) and Central Zones (60.9%) but remained > 7% point for North Zone (55.2%) (Table 2). There was a greater difference in corrected screening participation between North and Edmonton: 10 percentage points, and Calgary:11 percentage point among those aged 50–66 years.

**Table 2:** Hysterectomy corrected and uncorrected screening participation by zone of residence, migration status and ethnicity

	25–49 years			50–66 years		
Variables	Screening participation (%uncorrected)	Screening participation (%Corrected)	Difference (% point)	Screening participation (%uncorrected)	Screening participation (%Corrected)	Difference (%point)
Zone of residence						
South	58.2	61.4	3.2	49.5	58.3	8.8
Calgary	60.5	61.8	1.4	56.0	61.2	5.1
Central	57.8	60.9	3.1	48.3	56.2	7.8
Edmonton	61.3	62.9	1.6	54.8	60.7	5.9
North	53.0	55.2	2.2	44.0	49.8	5.8
Immigration status						
International immigrants	58.0	58.8	0.7	49.6	52.1	2.5
Inter-provincial immigrants	54.3	55.8	1.5	45.1	48.7	3.6
Non-immigrants	64.5	67.2	2.8	56.4	64.5	8.2
Ethnicity						
White	59.9	62.0	2.0	53.0	59.6	6.6
Black	58.5	60.2	1.7	51.2	57.6	6.4
Hispanic	61.8	63.1	1.3	55.3	60.1	4.8
Asian	57.0	57.6	0.6	54.4	57.5	3.2
Others	56.5	57.5	1.0	49.6	55.2	5.5

*N/A* Not Applicable

Screening participation (%) refers to proportion that were currently upto date screened

### Disparities based on immigration status

The corrected CCSPR based on immigration status revealed that non-immigrants had higher cervical cancer screening participation compared to both international and inter-provincial migrants (Table 2). Among those aged 25–49 years, inter-provincial migrants (55.8%) and international migrants (58.8%) had approximately, 8 and 11 percentage point lower screening participation than non-immigrants (67.2%), respectively. Greater differences (12 and 16 percent point) were found in those aged 50–66 years.

### Ethnic disparities

Using surname-based predicted ethnicity, we found Asian population (corrected: 57.6%) and the Black population (corrected: 60.2%) showed a lower screening participation compared to the White (corrected: 62.0%) among those aged 25-49 years. The difference in screening participation between Asian and White ethnicity increased from 2% point before correction to more than 4% after correction (Table 2).

### Multivariable Regression Results

Multivariable poisson regression analysis was conducted to study variation in screening participation. Our analysis confirmed disparities across the main predictor variables.

- Age: Women aged 30–39 (PR: 1.050, 95% CI: 1.044–1.056) and 40–49 years (PR: 1.056, 95% CI: 1.051–1.062) were more likely to participate in screening compared to those aged 25–29 years (Table 3). Whereas women aged 60–66 years were significantly less likely to participate in screening (PR:0.884, 95% CI: 0.880–0.889) compared to those aged 50–59 years.
- Geography: Women from the North zone were significantly less likely to participate in screening than women from Calgary zone in both 25–49 years (PR: 0.975, 95% CI: 0.962–0.987) and 50–66 years (PR: 0.866, 95% CI: 0.851–0.882). Women from the Central zone were significantly more likely to participate in screening among those aged 25–49 years and were significantly less likely to participate in screening among aged 50–66 years.
- Migration status: International (PR: 0.873, 95% CI: 0.869–0.877) and Interprovincial immigrants (PR: 0.832, 95% CI 0.829–0.836) aged 25–49 years were less likely participate in screening compared to non-immigrants. A similar pattern was found in those aged 50–66 years.
- Race/ethnicity: The Black (PR: 0.988, 95% CI 0.98–0.996) and the Asian population (PR: 0.944, 95% CI 0.939–0.950) were significantly less likely to participate in screening compared to the White population. Whereas the Hispanic population (PR: 1.043, 95% CI 1.033–1.052) were significantly more likely to participate in screening compared to the White population.

**Table 3:** Prevalence ratios for corrected screening participation using modified poisson regression

	25–49 years*			50–66 years*		
Variables	Screening participation prevalence ratio	95% CI	*p*-value	Screening participation prevalence ratio	95% CI	*p*-value
Age category						
25–29	ref			N/A	N/A	N/A
30–39	1.050	1.044–1.056	< 0.0001	N/A	N/A	N/A
40–49	1.056	1.051–1.062	< 0.0001	N/A	N/A	N/A
50–59	N/A	N/A	N/A	ref		
60–66	N/A	N/A	N/A	0.884	0.880–0.889	< 0.0001
Zone of residence						
Calgary	ref			ref		
South	1.032	1.018–1.046	< 0.0001	0.994	0.976–1.013	0.583
Central	1.034	1.021–1.047	< 0.0001	0.963	0.948–0.979	< 0.0001
Edmonton	0.990	0.986–0.994	< 0.0001	0.983	0.977–0.989	< 0.0001
North	0.975	0.962–0.987	0.0001	0.866	0.851–0.882	< 0.0001
Immigration status						
Non-immigrants	ref			ref		
International immigrants	0.873	0.869–0.877	< 0.0001	0.772	0.765–0.779	< 0.0001
Inter-provincial immigrants	0.832	0.829–0.836	< 0.0001	0.751	0.745–0.756	< 0.0001
Ethnicity						
White	ref			ref		
Black	0.988	0.980–0.996	0.004	0.986	0.973–0.999	0.0068
Hispanic	1.043	1.033–1.052	< 0.0001	1.079	1.062–1.097	< 0.0001
Asian	0.944	0.939–0.950	< 0.0001	1.001	0.992–1.010	0.730
Others	0.931	0.897–0.967	0.0002	0.945	0.885–1.008	0.086

*N/A*: Not Applicable

^*^Multivariable model includes variables in the table, social and material deprivation indices, and urban/rural place of residence

## Discussion

### Key findings and interpretation

In this large population-based study, we found that CCSPR increased after correcting for hysterectomy prevalence. Furthermore, the impact of correction varied significantly by age, with the largest increase observed among women aged 50–59. Prior to correction, participation appeared to decline with age after 30–39 years; however, hysterectomy correction revealed a stable CCSPR between ages 30 and 60, with a drop only in the 60–66 age group. These findings reinforce the importance of excluding ineligible women from the denominator to avoid underestimation of screening coverage, particularly in older age groups where hysterectomy prevalence is high. However, it is to be noted that full medical history and therefore hysterectomy data were incomplete for individuals > 50 years, therefore we observed only a modest increase upon correction in overall population and specifically among those > 50 years.

Our results showing reduced age disparity after correction for hysterectomy are consistent with past studies [7, 9, 27]. Forte et al., 2012 reported that CCSPR was maximum in 20–29-year-olds and declined with age before correction [9]. However, upon correction, there was consistent participation across age categories until the age of 60. In a Danish Study, Lam et al. found that uncorrected CCSPR declined after the age of 44 years. Upon correction, CCSPR slightly increased between the age of 30 and 60 years, after which it showed a decrease [27]. Unlike most of the earlier research relying on self-reported hysterectomy status or outdated survey data, our study used comprehensive administrative records and time-specific coding, allowing more accurate hysterectomy identification.

Despite correction, CCSPR remained below 65% across all age groups, underscoring persistent gaps in screening coverage. Of particular concern is the lower participation among women aged 60–66. One of the reasons for the apparent lower CCSPR is incomplete hysterectomy data in this age group. However, corrected CCSPR using self-reported data from CCHS survey also showed that CCSPR declined among > 65 years after reaching maximum in 60–64 years (Additional file 3). This group, nearing the upper age limit for routine screening, may be exiting screening without adequate prior testing. Evidence from the U.S. shows rising cervical cancer incidence among women over 65, possibly due to persistent new infections, reactivation of HPV, increased life expectancy, and reduced hysterectomy rates [6, 28]. Therefore, public health efforts should emphasize the importance of a negative screening history not just the chronological age before stopping screening. HPV primary testing may be a promising strategy for re-engaging under-screened older women.

Women aged 25–29 years also showed a lower CCSPR compared to those aged 30 years and above. This population has < 1% hysterectomy prevalence therefore hysterectomy correction has a minimal impact on estimated CCSPR. These women may face other barriers including low perceived risk (due to HPV vaccination) [29], limited awareness, stigma, or lack of access to youth-friendly services [30]. Tailored messaging and service models for younger populations remain essential.

The North Zone continued to show low CCSPR even after correction compared to Edmonton and Calgary, although much of the disparity in the Central and South Zones apparently disappeared upon correction. North Zone had a lower hysterectomy rate compared to other rural zones. Based on these findings it is imperative to implement additional targeted measures to improve CCSPR among women from North Zone. Successful interventions piloted in Alberta’s North Zone for integrating cervical cancer screening with breast and colorectal cancer screening programs through mobile outreach clinics can be expanded to wider geography and remote areas [31].

Similarly, immigrant characteristics and race/ethnicity showed a continuing disparity even after correction. Alberta has experienced increasing positive net migration in recent years, driven by both international and interprovincial migration [32]. Official data confirms that these trends are reshaping the province’s demographic landscape. Since migration patterns directly or indirectly affect the delivery and uptake of screening services, they may influence the estimated participation rates. Therefore, it is important to take migration factors (migration status, time since migration) into account while estimating CCSPR. Considering low screening participation in sub-populations based on migration and ethnicity warrants that targeted interventions need to consider these factors along with age of the target population.

It is interesting to note that interprovincial immigrants have lower screening participation rate compared to international immigrants. We initially expected that inter-provincial immigrants would have comparable screening rate to non-immigrants after excluding individuals who lived in Alberta for less than 3 years before the index date. However, they continued to show lower screening participation rate. One of the reasons may be due to lack of structured settlement and navigation support to interprovincial immigrants compared to international immigrants and refugees [33]. Potential misclassification of immigrant category may also contribute, because significant proportion of inter-provincial immigrants are newcomers or international immigrants who landed in other provinces before relocating to Alberta. Additional contributing factors include: less familiarity with Alberta’s cervical cancer screening program, inconsistent access to regular primary care providers and socio-economic or geographic challenges. In the light of these findings, Screening Programs need to consider interprovincial and international immigrants as a priority population in their program planning.

Hysterectomy rate is declining in Canada [12, 34]. Decreasing population level hysterectomy exposure will increase the number of women at risk of cervical cancer and consequently cervical cancer incidence, if screening rates remain sub-optimal. Since impact of HPV vaccination will not be seen in women > 60 years for decades, increase in cervical cancer incidence might partially counteract the anticipated decrease in cancer incidence due to organized cancer screening and other preventive strategies [35]. Therefore, any future interventions for cervical cancer prevention need to consider the impact of changing hysterectomy trend [35].

### Recommendations and future directions

Monitoring hysterectomy trends and their influence on CCSPR is essential to inform population-level cancer prevention strategies. Future studies can utilize more complete hysterectomy data to generate more reliable hysterectomy prevalence estimates for the > 50-year-olds. We anticipate that the CCSPR would further increase with more complete hysterectomy data. Data sharing initiatives across jurisdictions can help to capture complete hysterectomy picture for immigrants.

Given inequities across sub-populations, targeted interventions are critical to improve CCSPR. Public health messaging and patient education need to strongly reinforce the importance of adequate negative screening history (3 consecutive negative tests in the past 10 years) before screening can be stopped instead of emphasis solely on chronological age [36]. Younger age women need tailored messaging highlighting potential benefits of screening and potential harms of underscreening. Given lower health literacy, access and cultural barriers, tailored strategies to address unique needs of immigrant population will be equally important to improve screening uptake [17, 37, 38].

With falling hysterectomy incidence, cervical cancer prevention efforts need to take into account expanding populations at risk. Focusing on sub-populations with traditionally high hysterectomy prevalence to assess future impact and design potential interventions will be critical since the impact of hysterectomy correction on estimated CCSPR will decrease with falling prevalence of hysterectomy.

### Strengths and limitations

We used health administrative data to identify women who had a hysterectomy and to identify women who received pap tests. To our knowledge, this is the first Canadian study using province wide eligible population to estimate corrected CCSPR using population-based administrative data. Use of routinely collected health administrative data on health service utilization reduces selection bias and recall biases compared to self-reported data. Additionally, hysterectomy (total vs sub-total) and proxy indications for hysterectomy (benign vs history of high-grade lesion) were ensured using the relevant codes and predetermined definitions. Furthermore, we used administrative data to identify immigration status and used a robust and validated surname method to identify ethnicity of our participants. By reducing selection bias and measurement bias the study improved internal validity of measuring CCSPR. We used descriptive statistics (average uncorrected CCSPR, average corrected CCSPR, difference between corrected and uncorrected CCSPR) to compare corrected CCSPR across population categories and used inferential statistics (modified-poisson regression) to confirm those findings. Since we included province wide eligible population in the current analysis, the findings are generalizable to the Alberta population. ACCSP has introduced provider-led HPV testing for women aged 50–69 in November 2025 as a part of its cervical cancer screening program. Although screening interval to determine uptodate status for women aged 50–69 years will change, the hysterectomy correction method applied in this study remains applicable under the revised screening model.

Some of the limitations are worth mentioning. We studied administrative data over the past 25 years to estimate cumulative hysterectomy prevalence. Changes in procedure and billing codes overtime in Alberta during the look back window (1994–2019) may have caused misclassification of the procedure. However, we used time specific codes to minimize the misclassification. Any remaining misclassification is likely to underestimate hysterectomy prevalence and consequently underestimate CCSPR. Despite 25 years coverage, data was incomplete for those aged > 50 years and immigrants, resulting in potentially underestimated CCSPR for these groups. The study timeframe (i.e. 2019–2022) overlapped with COVID-19 pandemic, because during the planning phase of the project, the latest screening data was available only up to December 2022. Although the COVID-19 pandemic affected both screening utilization and hysterectomy uptake during the study period, we used hysterectomy prevalence data up to July 2019 to correct screening uptake which has no impact of the pandemic. We used proxy definition to identify hysterectomy conducted for benign reasons due to data unavailability. As a result, women who have had total hysterectomy due to high grade lesion may have been misclassified who should continue to get screened. Potential exclusion of such women from the denominator might have contributed to overestimation of CCSPR. However, we expect such impact to be minimum. Future studies should use more objective clinical measures (abnormal pathology/history of high-grade lesion) to avoid possible misclassification.

## Conclusion

This study provides critical insights into cervical cancer screening participation in Alberta, highlighting key measurements and programmatic considerations for improving accuracy in cervical cancer screening surveillance. This study provides insight into where disparities exist in eligible population after removing hysterectomized women. Hysterectomy correction narrowed age-wise disparity in CCSPR, although women aged 25–29 years and 60–66 years persistently showed lower screening participation. North zone of residence, migration status (international and inter-provincial migration), and ethnicity (Asian and Black) are other important factors to consider while designing targeted interventions to improve screening uptake.

## Supplementary Information

Additional file 1. Additional Figure 1.Data linkage procedure

Additional file 2. Additional Table 1a. Total hysterectomy CCI intervention codes (DAD, NACRS). Additional Table 1b. Total hysterectomy (Physician billing codes)

Additional file 3. Additional Table 2. Uncorrected and corrected estimates using CCHS 2013-2014

## Data Availability

The stewards of the data used in this study are Alberta Health Services and Alberta Ministry of Health, who maintain the data for the purpose of health system administration. Thus, the authors are not at liberty to make the data publicly available.

## Abbreviations

ACCSP: Alberta Cervical Cancer Screening Program
AHCIP: Alberta Health Care Insurance Population Registry
CCSPR: Cervical cancer screening participation rate
CUTD: Currently uptodate
DAD: Discharge abstract database
HPV: Human papilloma virus
NACRS: National ambulatory care reporting system
PR: Prevalence ratio
