# Supplementary figures and images for: Reduced age-wise disparity in estimated cervical cancer screening participation rates after applying hysterectomy correction: a population-based cross-sectional study
Source: BMC Womens Health. 2026 Mar 26;26:230. doi: 10.1186/s12905-026-04414-1 (PMC13141331; doi:10.1186/s12905-026-04414-1)

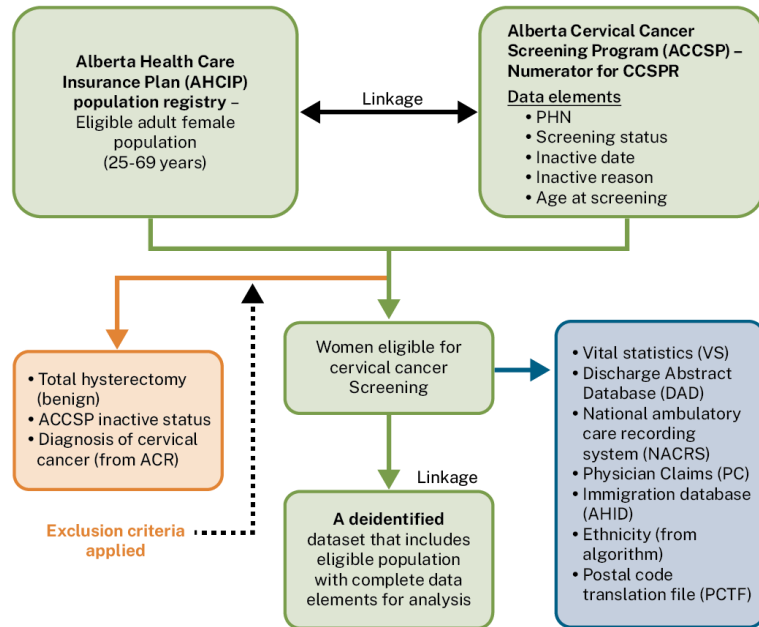

Additional Figure 1: Data linkage process

Supplement: Supplementary file 1 — Additional file 1. Additional Figure 1.Data linkage procedure [file 12905_2026_4414_MOESM1_ESM.pdf]
