# Supplementary material for: Reduced age-wise disparity in estimated cervical cancer screening participation rates after applying hysterectomy correction: a population-based cross-sectional study
Source: BMC Womens Health. 2026 Mar 26;26:230. doi: 10.1186/s12905-026-04414-1 (PMC13141331; doi:10.1186/s12905-026-04414-1)
Supplement: Supplementary file 2 — Additional file 2. Additional Table 1a. Total hysterectomy CCI intervention codes (DAD, NACRS). Additional Table 1b. Total hysterectomy (Physician billing codes) [file 12905_2026_4414_MOESM2_ESM.pdf]

Additional Table 1a. Total hysterectomy CCI intervention codes (DAD, NACRS)

Code	Definition
1RM89AA	Excision total, uterus and surrounding structures using combined laparoscopic and vaginal approach
1RM89CA	Excision total, uterus and surrounding structures using vaginal approach
1RM89DA	Excision total, uterus and surrounding structures using endoscopic (laparoscopic) approach
1RM89LA	Excision total, uterus and surrounding structures using open approach
1RM91AA	Excision radical, uterus and surrounding structures using combined laparoscopic and vaginal approach
1RM91CA	Excision radical, uterus and surrounding structures using vaginal approach (e.g. Schauta operation)
1RM91DA	Excision radical, uterus and surrounding structures using endoscopic (laparoscopic) approach
1RM91LA	Excision radical, uterus and surrounding structures using abdominal approach (e.g. Wertheim operation)
5MD60RC	Cesarean hysterectomy with use of forceps
5MD60RD	Cesarean hysterectomy with use of vacuum
5MD60KE	Cesarean hysterectomy without instrumentation
5MD60CB	Cesarean section, with use of both vacuum and forceps cesarean hysterectomy
5.CA.89.GB	Surgical termination of pregnancy with hysterectomy (endoscopic approach)
5.CA.89.WJ	Surgical termination of pregnancy with hysterectomy (open approach)
5.CA.89.CK	Surgical termination of pregnancy with hysterectomy (vaginal approach)

Additional table 1b. Total hysterectomy (Physician billing codes)

Current Codes	Definition
81.99A	Hysterectomy, any method
86.9B	Cesarean hysterectomy
81.99C	Laparoscopic radical hysterectomy and bilateral radical lymph node dissection
Historical billing codes (1994-2002)	
80.3	Total abdominal hysterectomy
80.4	Vaginal hysterectomy
80.5	Radical Abdominal hysterectomy
80.6	Radical Vaginal hysterectomy
