# Supplementary material for: Reduced age-wise disparity in estimated cervical cancer screening participation rates after applying hysterectomy correction: a population-based cross-sectional study
Source: BMC Womens Health. 2026 Mar 26;26:230. doi: 10.1186/s12905-026-04414-1 (PMC13141331; doi:10.1186/s12905-026-04414-1)
Supplement: Supplementary file 3 — Additional file 3. Additional Table 2. Uncorrected and corrected estimates using CCHS 2013-2014 [file 12905_2026_4414_MOESM3_ESM.pdf]

Additional Table 2. Uncorrected and corrected estimates for cervical cancer screening participation using hysterectomy prevalence based on CCHS 2013-2014

	50-69 years		
Variables	Screening participation (uncorrected)	Screening participation (Corrected)	Difference
**Age category**			
50-59	54.9	66.38	11.48
60-69	50.1	68.49	18.39
**Zone of residence**			
South	49.53	67.9	18.37
Calgary	56.04	69.1	13.06
Central	48.31	64.6	16.29
Edmonton	54.84	68.2	13.36
North	44.03	57.8	13.77
**Immigration status**			
International immigrants	49.6	58	8.4
Inter-provincial immigrants	45.1	58.5	13.4
Non-immigrants	56.4	73.1	16.7
**Ethnicity**			
White	53	68	15
Black	51.24	61.4	10.16
Hispanic	55.25	66.2	10.95
Asian	54.38	65.2	10.82
Others	49.63	59.5	9.87
